# Preparation of Chitosan Oleogel from Capillary Suspension and Its Application in Pork Meatballs

**DOI:** 10.3390/gels10120826

**Published:** 2024-12-14

**Authors:** Shishuai Wang, Zhongqin Fan, Xinya Huang, Yue Gao, Hongwei Sui, Jun Yang, Bin Li

**Affiliations:** 1 College of Food Science and Technology, Wuhan Business University, Wuhan 430056, China; 2 College of Food Science and Technology, Huazhong Agricultural University, Wuhan 430070, China

**Keywords:** chitosan oleogels, capillary bridges, rheology, oil loss, hardness

## Abstract

In the oil dispersion of chitosan, the formation of a capillary bridge was triggered by adding a small amount of water to obtain an oleogel. With this method, the types of liquid oil and the ratio of oil/chitosan/water were explored to achieve an optimal oleogel. MCT performed best, followed by soybean oil, which was chosen for its edibility and cost. Increasing chitosan from 15% to 45% reduced oil loss from 46% to 13%, and raising the water/chitosan ratio from 0 to 0.8 lowered oil loss from 37% to 13%. After normalization, the optimal soybean oil, chitosan, and water ratio was 1:0.45:0.36, yielding a solid-like appearance, minimal oil loss of 13%, and maximum gel strength and viscosity. To assess the potential application of the optimized oleogel, it was incorporated into pork meatballs as a replacement for pork fat. Textural and cooking experiments revealed that as the oleogel content increased, the hardness of the pork meatballs increased, while the cooking loss decreased. It suggested that the chitosan oleogel could enhance the quality of pork meatballs while also contributing to a healthier product by reducing saturated fat content.

## 1. Introduction

Solid plastic fats, such as margarine and butter, are crucial in bakery food due to their desirable properties. However, they also bring an amount of saturated and trans fat into foods [1]. Modern nutrition has pointed out that the excessive consumption of these fats has a negative impact on health, increasing the risk of type 2 diabetes, cardiovascular diseases, obesity, and other diseases [2,3]. Oleogels, as a promising oil structuring system, have the advantage of retaining the nutritional value of liquid oils without altering their chemical composition while providing the texture of solid fats [4]. Therefore, the development of oleogel is essential for the reduction or replacement of saturated fat and trans fat in food products [5].

At present, techniques of oleogel preparation, which include direct methods based on gelling agents, polymer self-assembly, or crystallization, as well as indirect methods based on emulsion templates, have exhibited certain deficiencies in industrial applications [6,7]. These deficiencies are primarily characterized by complex procedures, stringent conditions, and prolonged processing times, which limit their scalability and efficiency [8]. Enzymatic interesterification, as another technique for altering the characteristics of oil and fat blends, rearranges fatty acids within the glycerol backbone to form structured oleogels [9]. Compared to chemical processes, enzymatic interesterification offers several advantages, including operation under mild conditions, reduced energy consumption, enhanced lipid quality, and decreased by-product generation. However, the method faces challenges such as high costs associated with biological enzyme preparations and production expenses, which restrict its feasibility in industrial-scale production [10]. Furthermore, bigels, a new type of biphasic gel fat substitute containing both aqueous and oily phases [11], require the consideration of multiple factors in their preparation, including gelling agents, oil/hydrogel ratios, lipid properties, and preparation processes. The precise control of these factors necessitates specialized equipment and technicians, thereby increasing the complexity and cost of the preparation process. Additionally, the types of gels used in food are limited, and high-quality raw materials are often expensive and relatively scarce in the market, further increasing the cost of bigels [12].

Currently, a novel approach to fabricating oleogel via capillary suspension has attracted wide attention due to its environmentally friendly, user-friendly, and efficient nature, along with its ease of industrial application and tunable properties. Capillary suspension, which involves successively dispersing particles and water (secondary fluid) in liquid oil (primary fluid), induces a capillary liquid bridge between particles to form oleogel [13]. In the process, the liquid oil can be converted into a solid fat- or gel-like state without any changes in chemical properties. Notably, the process is simple and eco-friendly, as it does not require the use of any chemical reagents [14]. Unlike traditional margarine and butter, liquid oils such as soybean oil are rich in about 80% unsaturated fat. Therefore, the plastic fat prepared by the ternary combination of liquid oil, water, and particles can effectively reduce the content of saturated and trans fat, and become a low-fat health food that is friendly to consumers [15].

The properties of capillary suspension are affected by a number of factors, including the volume fraction of particles, the particle radius, the coordination number (the number of liquid bridges connected to each particle), the volume of the capillary liquid bridges, the interfacial tension between two phases, and the mutual wettability of the ternary system [16,17]. The complex relationship between these factors has been revealed by mathematical equations [18,19]. However, academic articles on capillary suspension in food have still been relatively scarce. Up to now, only a dozen or so studies have been reported involving corn starch [20,21,22], cellulose [23,24], zein [25], cocoa granules [26], soybean fiber [27], pea fiber [28], wheat bran [29], tomato peel, and coffee grounds [30]. Therefore, it is particularly important to explore new food resources to prepare capillary suspensions and evaluate their application potential in food.

Chitosan, a deacetylated derivative of chitin, is predominantly sourced from the shell of crustaceans such as shrimp and crabs. As the sole natural alkaline cationic polysaccharide, chitosan exhibits excellent biocompatibility, biodegradability, and adsorptive properties [31,32]. In the food industry, chitosan is used in a wide range of applications, including as a preservative to prolong food shelf life, the fabrication of edible packaging materials, the enhancement of food consistency, the promotion of emulsification, the delivery of bioactive ingredients, and the improvement of cooking characteristics [33,34,35,36]. However, the preparation of capillary suspension by chitosan powder and its application has not been reported.

Herein, chitosan served as the main material to fabricate oleogel via capillary suspension. The properties of chitosan oleogel were optimized by screening the types of liquid oil and adjusting the ratio among chitosan, oil, and water. Additionally, the appearance, oil loss, and rheological properties of oleogel were considered key evaluation indicators. Furthermore, the application effect of chitosan oleogel on pork meatballs was assessed based on the texture and cooking loss, thereby contributing to the quality improvement of pork meatballs.

## 2. Results and Discussion

### 2.1. Optimization of Chitosan Oleogel

#### 2.1.1. Influence of Liquid Oil Type on Oleogel Properties

**Figure 1:**
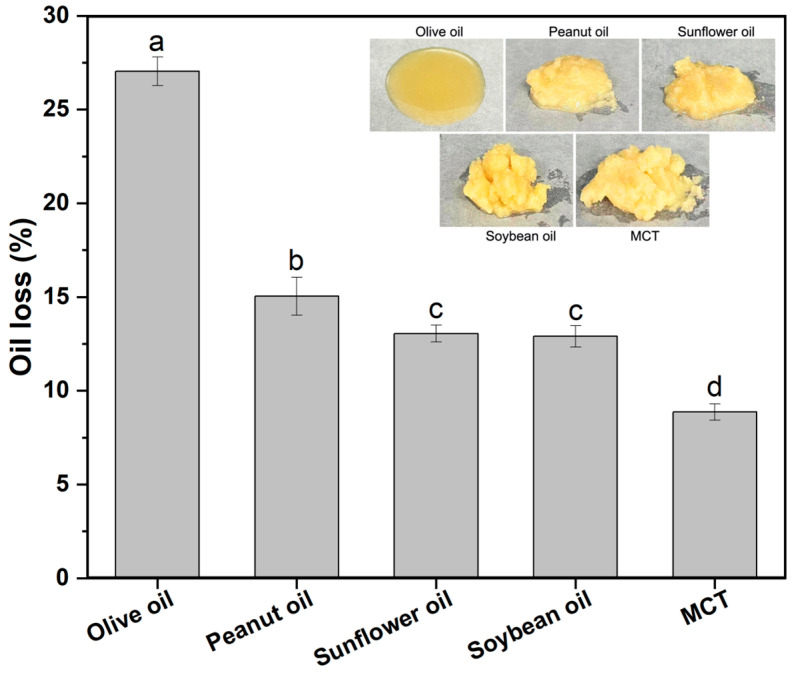
Oil loss of chitosan oleogels made with different liquid oils, embedded with the corresponding oleogel pictures. Different letters denoted significant difference (*p* ≤ 0.05).

To investigate the impact of liquid oil type on oleogel properties, five types of oils were selected, including four commonly consumed edible oils—soybean oil, peanut oil, sunflower oil, and olive oil—as well as medium-chain triglycerides (MCTs). Throughout the experiment, the mass ratio of oil/chitosan/water was constant at 1:0.45:0.36. As illustrated in Figure 1, among the four edible oils, the oleogel formulated with soybean oil demonstrated superior oil-holding capacity with a relatively low oil loss of 13%. Conversely, the oleogel prepared with olive oil showed the worst performance, exhibiting the highest oil loss of 27%. Notably, the oleogel made with MCT significantly outperformed all four edible oils in terms of oil-holding capacity, achieving an oil loss of only 9%. The literature reported that oleogels that incorporated 6% carnauba wax into soybean oil and peanut oil maintained an oil binding capacity of about 80% when stored at 25 °C for a day [37]. This observation suggested that the oil-holding capacity of chitosan-based capillary suspensions was comparable to that of wax-based oleogels. The visual inspection of the oleogel photographs revealed that the oleogel prepared with olive oil exhibited a tendency to flow, whereas the oleogels prepared with the other oils possessed inherent plasticity and were not prone to flowing.

**Figure 2:**
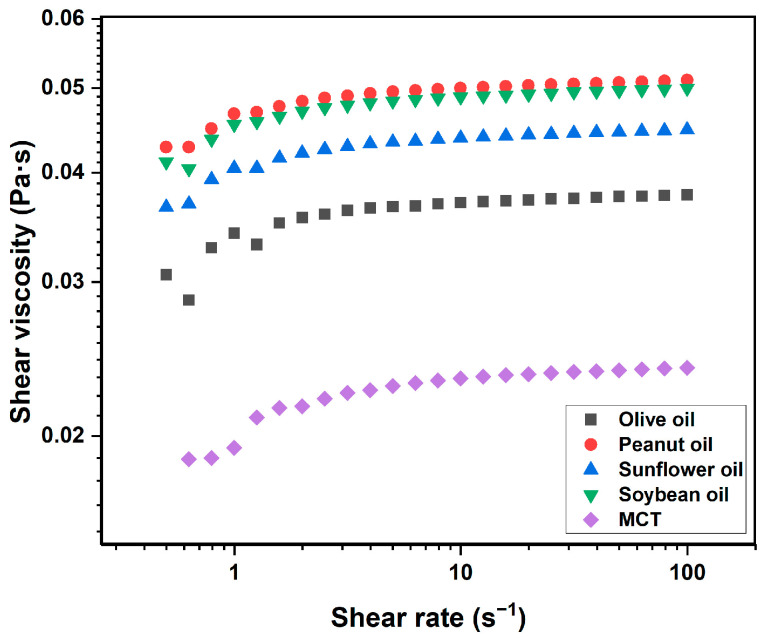
Shear viscosity of five pure liquid oils.

Considering a potential association between the oil-holding capacity of the oleogel and liquid oil viscosity, the viscosity of a single liquid oil was measured, as presented in Figure 2. The viscosities of the liquid oils were ranked as follows: peanut oil > soybean oil > sunflower oil > olive oil > MCT. Despite the lowest viscosity of MCT, the oleogel prepared from it exhibited the maximal oil-holding capacity. Among the other four edible oils, no correlation was observed between the oil viscosities and oil retentions of the respective oleogels. Specifically, the oleogel formed from olive oil, which had the lowest viscosity, exhibited the poorest oil retention, whereas the oleogel prepared from soybean oil, whose viscosity was neither the highest nor the lowest, demonstrated optimal oil retention. Based on the phenomenon, it was speculated that the viscosity of liquid oil was not the sole determinant of the oil-holding capacity of the oleogel. Emulsification might be another factor affecting oleogel properties. It was discovered that the pre-emulsification process helped to form a more uniform and stable structure of the oleogel, which contributed to the excellent oil-holding capacity of capillary suspension [38]. Phospholipids and soybean protein, as natural emulsifiers present in soybean oil, might play a significant role in the oil retention of the oleogel [39].

**Figure 3:**
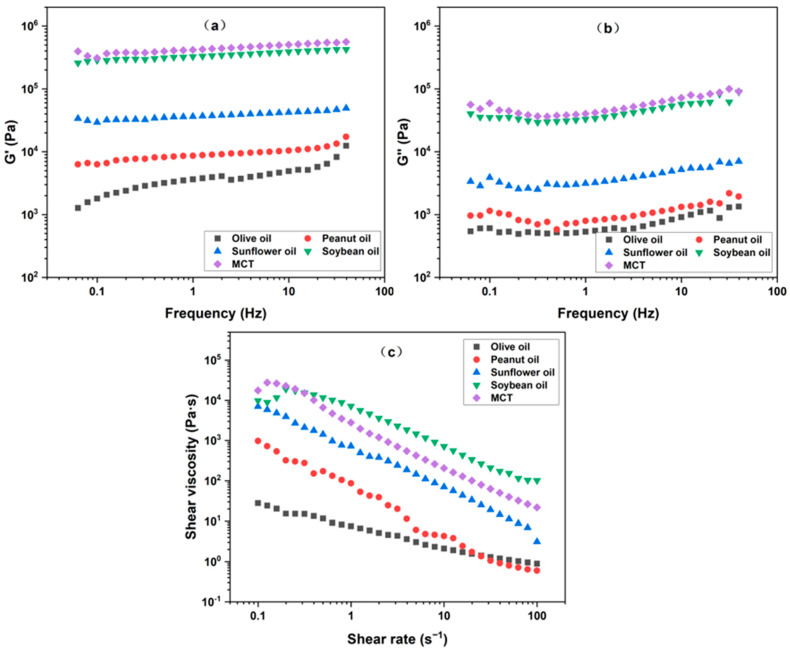
Rheological curves of chitosan oleogel prepared from different liquid oils: (**a**) storage modulus G′ vs. frequency; (**b**) loss modulus G″ vs. frequency; (**c**) shear viscosity vs. shear rate.

The effect of liquid oil type on the dynamic viscoelastic properties of oleogels is illustrated in Figure 3a,b. The storage modulus (G′) of all the samples significantly exceeded the loss modulus (G″), with G′ being approximately ten times larger than G″. This observation indicated a solid-like gel state, suggesting that various liquid oils could form a network structure through capillary action in the presence of water and chitosan particles. In terms of gel strength, the oleogel formed with MCT had the highest G′, followed by the oleogel made with soybean oil, while the oleogel with olive oil presented the lowest G′. This trend coincided with the aforementioned trend in oil retention.

The type of liquid oil also affected the shear viscosity of the oleogel. As shown in Figure 3c, the shear viscosity of all the samples decreased with the increase in shear rate, showing the characteristics of pseudoplastic fluids. In addition, there were also differences in shear viscosity among the oleogel. Specifically, the viscosity of the oleogel formed by soybean oil was the highest, followed by that of MCT, while olive oil had the lowest viscosity. This might be related to the fact that the viscosity of pure soybean oil was higher than MCT (as seen in Figure 2), which contributed to the overall viscosity of the multicomponent oleogel. Taking into account usability and economic cost, soybean oil was selected as the next material.

#### 2.1.2. Influence of Chitosan Content on Oleogel Properties

**Figure 4:**
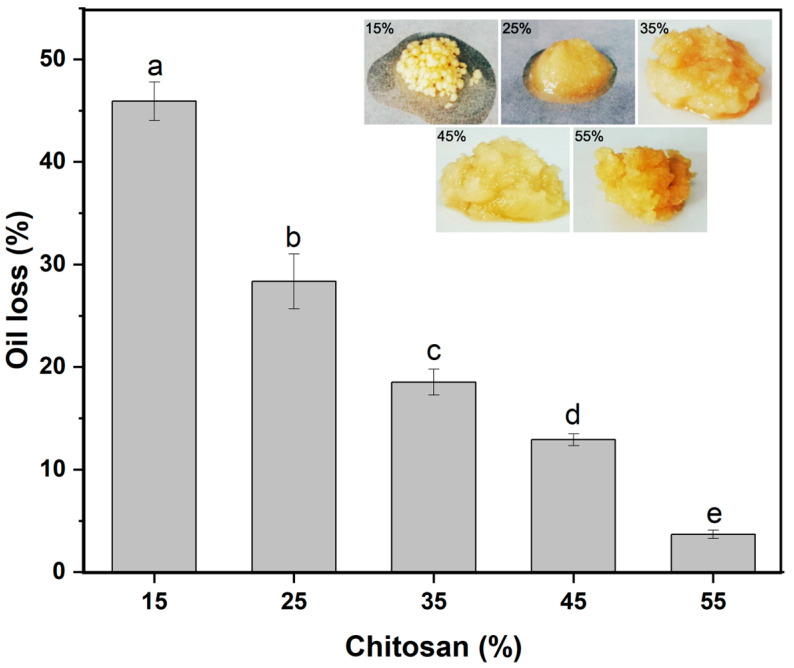
Oil loss of oleogels with different chitosan contents, embedded with the corresponding oleogel pictures. Different letters denoted significant difference (*p* ≤ 0.05).

The oleogel properties were influenced not only by the type of liquid oil but also by the composition ratio within the ternary system. To assess the impact of chitosan content on oleogel properties, the mass ratio of soybean oil to water was fixed at 1:0.36. The chitosan content was calculated by the ratio of chitosan mass to soybean oil mass. As illustrated in Figure 4, as the chitosan content rose from 15% to 55%, the oil loss in the oleogel significantly decreased, from an initial 46% down to merely 4%. Meanwhile, photos clearly showed that when the chitosan content was as low as 15%, the chitosan particles agglomerated and a large amount of liquid oil exuded. The leakage of liquid oil decreased with the increase in the chitosan content. It underscored that the elevation in chitosan content effectively enhanced the oil-holding capacity of the system, thereby imparting more stable oleogel for various applications.

**Figure 5:**
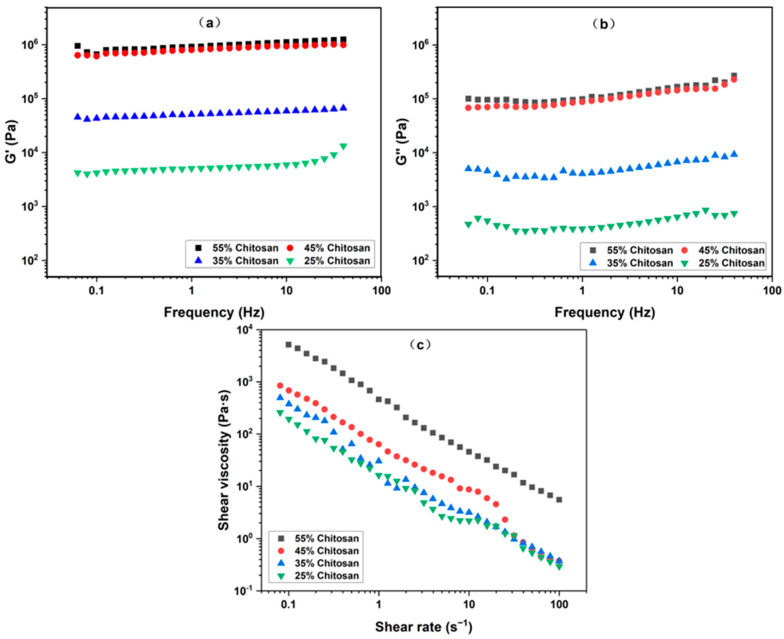
Rheological curves of oleogels with different chitosan contents: (**a**) storage modulus G′ vs. frequency; (**b**) loss modulus G″ vs. frequency; (**c**) shear viscosity vs. shear rate.

The impact of the chitosan content on the rheological properties of oleogel is illustrated in Figure 5a,b. In the entire range of frequency, both G′ and G″ of the samples slightly increased with rising frequency regarding a gel-like behavior. For the chitosan content to be depleted to 15%, obvious particle agglomeration and oil leakage occurred, resulting in a non-uniform system where the rheological properties were not accurately determined. A similar phenomenon was found in the capillary suspension based on corn starch [22]. Notably, as the chitosan content varied from 25% to 45%, there was a significant enhancement in both G′ and G″ of all the samples. This augmentation could be attributed to the increased number of capillary bridges formed between chitosan particles, thereby strengthening the gel network [21]. However, further incrementing the chitosan content to 55% did not result in a pronounced increase in the modulus. The reason was that an excessive amount of chitosan particles led to a more homogeneous distribution within the system. At the same time, the chitosan particles tended to aggregate and form self-supporting structures, which no longer constituted a gel network but rather particle clumps [20]. Consequently, the system transitioned from a gel network state to a non-networked state. Furthermore, in the presence of limited water, as the chitosan content increased, the number of water molecules surrounding the chitosan particles decreased. This reduction weakened the interactions between water and the chitosan particles, consequently leading to a decline in the gel strength [40].

Figure 5c clearly shows the effect of chitosan content on the shear viscosity of the oleogel. The viscosity of the system increased steadily as the chitosan particle content was raised from 25% to 45%. However, when the chitosan content further jumped to 55%, the viscosity increment was notably greater. This change was due to the excessive increase in the particle content in the system, which led to a significant increase in the interparticle friction, thus greatly improving the overall viscosity [41]. Consequently, a chitosan concentration of 45% was selected for further optimization.

#### 2.1.3. Influence of Water/Chitosan Ratio on Oleogel Properties

**Figure 6:**
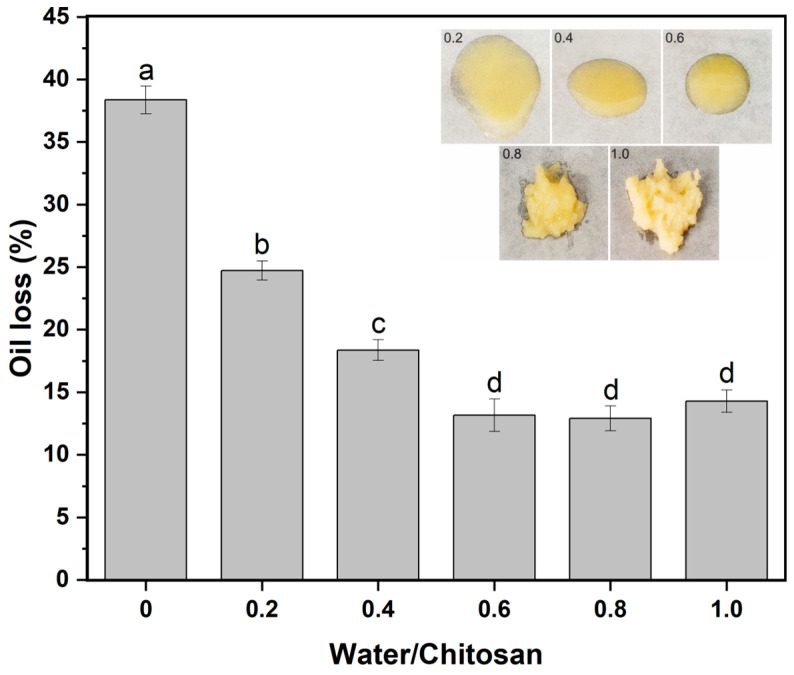
Oil loss of oleogels with different water/chitosan ratios, embedded with the corresponding oleogel pictures. Different letters denoted significant difference (*p* ≤ 0.05).

Although the water amount in the oleogel was relatively small in comparison to the other components (oil and chitosan), the oleogel properties were notably dependent on the minor component [42]. Throughout the experiment, the mass ratio of soybean oil to chitosan was fixed at 1:0.45. Oil loss of the oleogel varied with the water/chitosan ratio, as demonstrated in Figure 6. Initially, as the water/chitosan ratio increased from zero to 0.8, oil loss significantly declined from 37% to 13%. However, a further increase in the water/chitosan ratio to 1.0 led to a slight increase in oil loss. Consequently, it was concluded that the optimal oil retention of the oleogel was achieved at the water/chitosan ratio of 0.8. Excessive water led to extremely high viscosity in the system, making it challenging to achieve uniformity during the preparation [43]. In the unbalanced state, the network structure of the oleogel became weakened, resulting in a decrease in the oil-holding capacity. Additionally, observations from oleogel photographs revealed that the system tended to flow when the water/chitosan ratio ranged between 0.2 and 0.6. However, as the ratio increased to 0.8, the oleogel began to exhibit a solid form.

**Figure 7:**
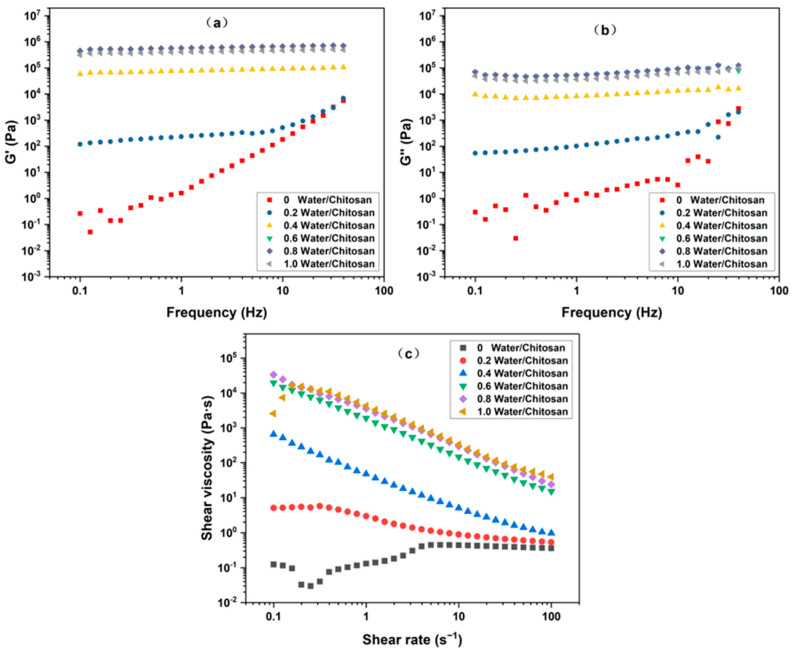
Rheological curves of chitosan oleogels with different water/chitosan ratios: (**a**) storage modulus G′ vs. frequency; (**b**) loss modulus G″ vs. frequency; (**c**) shear viscosity vs. shear rate.

Different water/chitosan ratios significantly impacted the dynamic viscoelasticity of the oleogels, as depicted in Figure 7a,b. When the water/chitosan ratio ranged from 0.4 to 1, the G′ and G″ of the system remained stable across the entire frequency range, indicative of a typical gel state. As the water/chitosan ratio increased from 0.4 to 0.8, the gel strength of the system gradually enhanced. However, when the ratio of water to chitosan reached 1.0, the gel strength of the system began to decline. This suggested that with an appropriate amount of water, sufficient capillary bridges could form between chitosan particles, significantly altering the rheological properties of the oleogel [17]. Insufficient water content led to a reduced number of capillary liquid bridges within the ternary system, along with insufficient hydrogen bonds between water molecules and particles [44]. Consequently, the oleogel formed under these conditions failed to achieve optimal properties, such as maximum oil retention and gel strength. Conversely, excessive water content facilitated phase separation, thereby reducing gel strength. So, by carefully adjusting the water/chitosan ratio, the strength of the oleogel could be effectively modulated.

Figure 7c clearly demonstrates the viscosity changes in the oleogels after the addition of water. As the water/chitosan ratio increased, the viscosity of the oleogels exhibited a rising trend. When the ratio increased from 0 to 0.8, the viscosity of the system underwent a notable rise. However, further increasing the ratio to 1.0 resulted in a continued but less significant increase in viscosity. This progression reflected the transformation of the internal network structure from a pendulum-like state to a capillary aggregation state [20].

In summary, the optimal preparation of oleogels involved a mass ratio of chitosan to soybean oil of 45%, and a water/chitosan mass ratio of 0.8:1. After normalization, the ideal mass ratio among soybean oil, chitosan, and water was 1:0.45:0.36.

**Figure 8:**
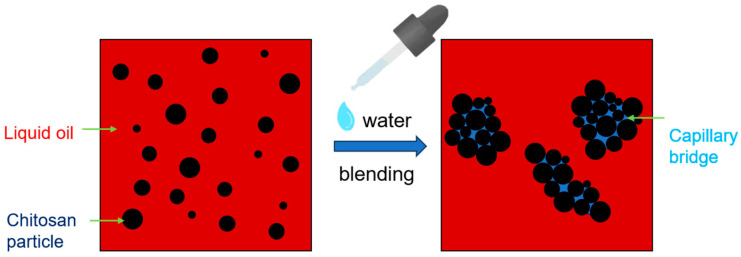
Schematic diagram of the formation mechanism of oleogel after incorporating water into the chitosan/liquid oil mixtures. The black circle represents chitosan particles, the red represents liquid oil, and the blue represents water.

To elucidate the pivotal role of water in the formation of a chitosan oleogel, Figure 8 depicts a schematic representation of a chitosan/liquid oil mixture both with and without the presence of water. In the absence of water, the chitosan particles failed to establish three-dimensional networks and were merely dispersed throughout the liquid oil. However, upon the introduction of water, capillary liquid bridges emerged between the particles, interconnecting them and endowing the mixture with solid-like characteristics. These capillary forces enhanced the cohesion among the particles within the oleogel, leading to a significant transformation in the rheological properties of the particles.

### 2.2. Application of Chitosan Oleogel in Pork Meatballs

#### 2.2.1. Hardness of Pork Meatballs

**Figure 9:**
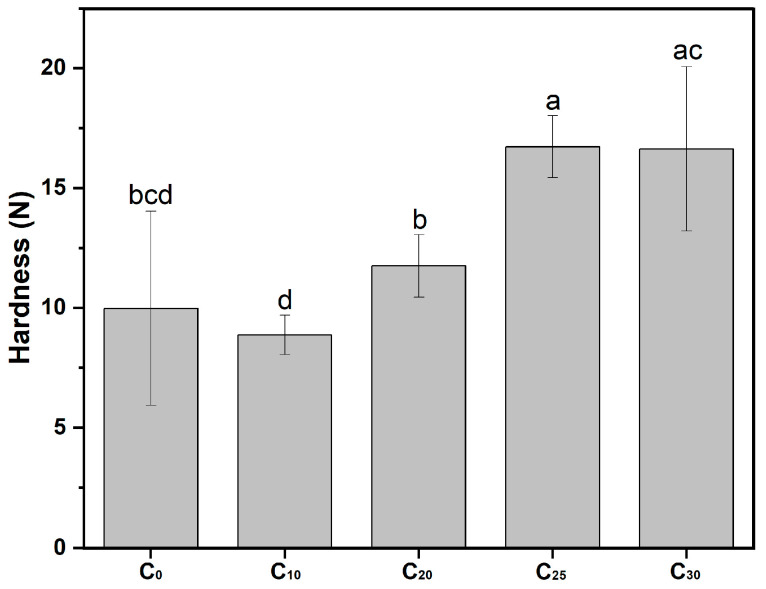
Hardness of pork meatballs with different proportions of chitosan oleogel. Different letters denoted significant difference (*p* ≤ 0.05).

Chitosan, as a type of dietary fiber, either alone or in combination, has been evaluated as a substitute for fat in meat products, aiming to improve health properties while giving meat products the desired texture [45]. Herein, the application of the optimized chitosan oleogel to pork meatballs was evaluated. The impact on the texture of pork meatballs is depicted in Figure 9. Compared to C_0_, which contained no oleogel, C_10_ exhibited a slight reduction in hardness, while C_20_ and C_25_ showed a gradual increase in hardness. It suggested that an appropriate amount of chitosan oleogel effectively enhanced the texture of meatballs, providing a more satisfying mouthfeel and improved chewiness. The finding was in agreement with Han et al. who reported that chitosan as a fiber promoted a firmer texture and might act as a binder, thereby favoring the formation of a stronger gel [45,46].

#### 2.2.2. Cooking Loss of Pork Meatballs

**Figure 10:**
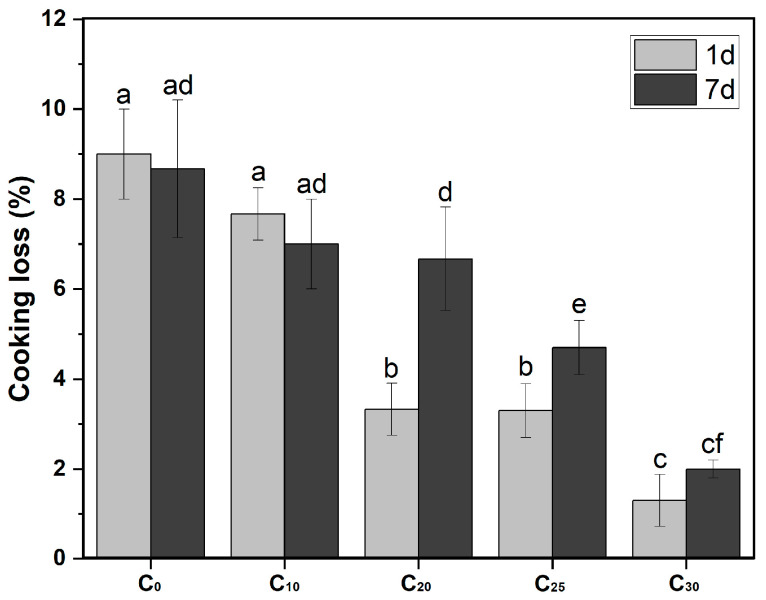
Cooking loss of pork meatballs with different proportions of chitosan oleogel for a storge of 1d and 7d. Different letters denoted significant difference (*p* ≤ 0.05).

After the pork meatballs were prepared, they were stored at 4 °C. Over the course of storage, extending from 1 to 7 days, the cooking loss of the pork meatballs increased, especially in C_20_, C_25_, and C_30_. This is a phenomenon likely linked to the proliferation of microorganisms. During their growth, these microbes broke down proteins, leading to a loosening of the meatball’s structural integrity. Additionally, protein oxidation that occurred during storage further intensified these changes, causing further damage to the original structure of the meatballs and diminishing their water-retaining capacity [47]. In addition, during storage, an increased content of chitosan oleogel in pork meatballs was correlated with a reduction in the fat content and a decrease in cooking loss. Notably, regardless of being stored for 1 day or 7 days, C_0_ without chitosan oleogel experienced the highest cooking loss, at nearly 9%. In contrast, C_30_ with the highest chitosan content exhibited the lowest cooking loss, below 2% (detailed in Figure 10). This change was primarily due to the properties of the chitosan oleogel.

The chitosan oleogel played a pivotal role in moisture retention within the meatballs. The gel structure effectively trapped water molecules, which existed in the capillary liquid bridge between chitosan particles. Upon heating, the proteins in the meatballs denatured, creating a compact network that encapsulated the oleogel and retained the water in the oleogel [7,48]. Consequently, as the chitosan oleogel content rose, it significantly minimized the cooking loss of the pork meatballs.

## 3. Conclusions

The properties of chitosan oleogel via capillary suspension, including appearance, oil retention, and rheology, could be regulated by the type of liquid oil or the ratio of chitosan to oil to water. The results showed that the chitosan oleogel prepared by MCT and soybean oil exhibited superior performance. Furthermore, the ratio of chitosan, soybean oil, and water was optimized to be 1:0.45:0.36. This specific composition yielded an oleogel with a solid fat phenotype, characterized by minimal oil loss and enhanced gel strength and viscosity. When incorporated as a pork fat substitute in meatballs, the chitosan oleogel not only increased the hardness but also reduced the cooking loss of the meatballs. These findings highlight the potential of chitosan oleogel as a healthier and more functional alternative to traditional fats in meat products.

## 4. Materials and Methods

### 4.1. Materials

Chitosan (a deacetylation degree of 90% and a viscosity of 200 mPa·s) was offered by Sinopharm Group Chemical Reagent Co., Ltd. (Beijing, China). Medium-chain triglycerides (MCTs) were purchased from Shanghai Yuanye Bio-Technology Co., Ltd. (Shanghai, China). Soybean oil, olive oil, peanut oil, and sunflower oil were obtained from commercial suppliers. Lean and fat pork were sourced directly from local supermarkets.

### 4.2. Preparation of Chitosan Oleogel

The chitosan powder was pre-dispersed in the liquid oil and stirred at a speed of 200 rpm for 20 min. Subsequently, the stirring speed was escalated to 500 rpm, and water was quickly added drop by drop. After thorough mixing, the chitosan oleogel was achieved. The preparation of the chitosan oleogel was optimized by adjusting the concentrations of chitosan and water, along with the selection of various liquid oils such as soybean oil, olive oil, peanut oil, sunflower oil, and MCT. The appearance of the chitosan oleogel was documented through photographs.

### 4.3. Measurement of Oil Loss from Chitosan Oleogel

Oil loss was determined using the following method [23]: The prepared chitosan oleogel was weighed and marked as m_1_. After centrifugation at 5000× *g* for 2 min, liquid oil precipitation was observed and its mass was recorded as m_2_. The oil loss can be calculated using the following formula: oil loss = m_2_/m_1_ × 100%.

### 4.4. Measurement of Rheological Properties of Chitosan Oleogel

The chitosan oleogel was tested on a controlled-stress Kinexus 2500 rheometer (Malvern Instruments Ltd., Worcestershire, UK) by a plate fixture with a 20 mm diameter. The frequency sweep was performed over a range of 0.01–100 Hz with a constant strain of 0.05%. Additionally, the viscosity of the oleogel was measured at shear rates ranging from 0.1 to 100 s^−1^. All the tests were conducted at a controlled temperature of 25 °C.

### 4.5. Preparation of Pork Meatballs with Chitosan Oleogel

The basic recipe for the pork meatballs consisted of the following: For every 100 g pork (70 g lean and 30 g fat), 3 g salt, 8 g starch, and 20 g water were added. To reduce the saturated fat content in pork meatballs, the chitosan oleogel was utilized as a replacement for the pork fat. Depending on the substituted quantity of fat, the pork meatballs were labeled as C_0_, C_10_, C_20_, C_25_, and C_30_ (detailed in Table 1).

### 4.6. Measurement of Hardness of Pork Meatballs

The hardness of pork meatballs was determined using the shear mode of a TMS/Pro Texture Analyzer (FTC Corporation, Sacramento, CA, USA) under the following conditions: trigger force of 0.5 N, test speed of 60 mm/min, return speed of 120 mm/min, and return distance of 35 mm.

### 4.7. Measurement of Cooking Loss of Pork Meatballs

The pork meatballs, initially weighing w_1_, were stored at 4 °C. After 1 day or 7 days of storage, they were heated in boiling water for 6 min, and then weighed again after the surface moisture was removed. The new weight was recorded as w_2_. The cooking loss of pork meatballs can be calculated using the following formula: cooking loss = (w_1_ − w_2_)/w_1_ × 100%.

### 4.8. Statistics Analysis

Data are presented as mean ± standard deviation. The figures were created using the Origin 2021 software (OriginLab Corporation, Northampton, MA, USA). Statistical analyses, including ANOVA to assess significant differences, were executed with the SPSS 21.0 software (IBM Corp., Armonk, NY, USA), with a *p*-value ≤ 0.05 indicating statistical significance. Each experiment was repeated three times at least.

## Figures and Tables

**Table 1:** Composition of pork meatballs (g).

Sample	Chitosan Oleogel	Fat Pork	Lean Pork	Salt	Starch	Water
C_0_	0	30	70	3	8	20
C_10_	10	20	70	3	8	20
C_20_	20	10	70	3	8	20
C_25_	25	5	70	3	8	20
C_30_	30	0	70	3	8	20

## Data Availability

The data presented in this study are openly available in GELS.
